# Less Is More: Genome Reduction and the Emergence of Cooperation—Implications into the Coevolution of Microbial Communities

**DOI:** 10.1155/2019/2659175

**Published:** 2019-02-17

**Authors:** Emanuele Bosi, Flavia Mascagni

**Affiliations:** ^1^Department of Biomedical Experimental and Clinical Sciences, University of Florence, Italy; ^2^Department of Agriculture, Food and Environment (DAFE), University of Pisa, Italy

## Abstract

Organisms change to adapt to the environment in which they live, evolving with coresiding individuals. Classic Darwinism postulates the primal importance of antagonistic interactions and selfishness as a major driver of evolution, promoting an increase of genomic and organism complexities. Recently, advancements in evolutionary ecology reshaped this notion, showing how leakiness in biological functions favours the adaptive genome reduction, leading to the emergence of codependence patterns. Microbial communities are complex entities exerting a gargantuan influence on the environment and the biology of the eukaryotic hosts they are associated with. Notwithstanding, we are still far from a comprehension of the ecological and evolutionary mechanisms governing the community dynamics. Here, we review the implications of genome streamlining into the unfolding of codependence within microbial communities and how this translates to an understanding of ecological patterns underlying the emerging properties of the community.

## 1. Introduction

In his 1862 book “*Fertilisation of Orchids*” [1], Charles Darwin postulated the coevolution of the orchid and fertilizing insects. Puzzled by the unusual length of the orchid *Angraecum sesquipedale* spur (around 30 cm long), Darwin predicted the existence of a pollinator moth with a proboscis nearly as long as the orchid spur (“…in Madagascar there must be moths with proboscides capable of extension to a length of between ten and eleven inches”). More than that, Darwin proposed a competition model to explain the emergence of such unusual features, according to which: (i) plants with longer spur are more easily fertilized by moths, since the insects have to delve deep in the flower to reach the nectar, resulting in a better impollination; (ii) insects with longer proboscis easily gather the plant's nectar with less energy dispersion, acquiring more nutrients at the expense of the plant's fertilization; (iii) plants with longer spurs are then positively selected; and (iv) the insects need longer proboscides to have an easy time feeding on the plant nectar. In other words, the outcome of such relationship established between these species is an arm race which favours individuals with increasingly long spurs/proboscides.

The concept that biotic interactions (such as the mutual competition reported above) are a major driver of evolution stands at the basis of the Red Queen (RQ) hypothesis [2]. Named after a quote from *Through the Looking-Glass*, “It takes all the running you can do, to keep in the same place.”, the original RQ is a macroevolutionary hypothesis proposing that coevolution of the interacting species might account for constant extinction rates observed in a number of taxa (opposed to sudden extinction caused by abiotic factors). On a microevolutionary level, RQH has been applied in the context of host-parasite interactions and in particular to explain the advantage of sexual reproduction [3, 4] over other reproductive strategies [5]. Indeed, the host-parasite interaction is ubiquitous and largely influenced by genetics, leading to frequency-dependent selection of genotypes. Therefore, genetic variability introduced by sexual reproduction would provide a substantial advantage facilitating the generation of novel/rare genotypes able to cope with the parasite infection [6].

Antagonistic interactions are not the only force pushing coevolution. Cooperation is pervasively diffused, if not inevitable, in nature. Looking at the biochemistry of different organisms, from eukaryotic hosts to small microbes, there are a number of compounds which cannot be synthetized but must be gathered from external sources (such as diet or symbionts). For instance, bacteria living in the human gut are auxotroph for different compounds which are acquired from the host, repaying it with vitamins (B_1_ and B_12_) and other metabolites having a positive impact on human health. The Black Queen (BQ) hypothesis [7] has been recently proposed to explain the evolutionary dynamics leading to such dependency, which is tightly connected to the concept of “leakiness.” In brief, a number of biological processes produce “leaky” goods that are available from other organisms. Therefore, genetic elements of these “beneficiaries” involved in such processes become dispensable and can be lost. Individuals undergoing such gene loss events will be advantaged and take over their population. The BQ interactions represent a force promoting an adaptive genome reduction, whose strength depends on a number of factors, including the overlap of ecological niches and the presence of other organisms contributing (or subtracting) these shared resources [8]. The BQ has been applied to simple prokaryotic systems, making possible a validation through laboratory (co)evolution experiments [9].

The routes leading to genome reduction, or simplification, which can be adaptive [7] and not merely the product of neutral gene loss [10], challenge the evolutionary view according to which life on earth is characterized by an increase of the complexity in time. Although genome complexity does not necessary scale with the (hard to define) organismal complexity [11], loss of genes in prokaryotes usually implies loss of functions. Therefore, this mode of evolution brings some functional constraints that must be fulfilled from the environment. In other words, the apparent conflict between evolutionary simplification and the “Zero Force Law of Evolution” [12], stating that unconstrained evolution leads to a monotonic increase in the average organismal complexity [13], is solved by thinking that the simplification in one organism is allowed by the complexity increase of the environment. Thus, the overall complexity of a biological system subject to reductive evolution increases since it now requires specific ecological interactions.

In this review, we will describe the implications of genome reduction in biological systems defined by complex ecological interactions, including microbial communities and holobionts, which are the combination of eukaryotic hosts and their microbiota.

## 2. Cooperation Is Pivotal for the Stability of Microbial Communities

Although classic microbiology emphasized the use of pure cultures, in nature, microbes are part of complex communities in which they interact with each other. These ecological interactions include not only “selfish” relationships like predation or competition but also synergistic [14] ones, such as syntrophy [15], protection against chemophysical stress [16, 17], and access to limited resources [18, 19]. Following the BQ nomenclature, functions whose products are (at least partially) shared with other organisms in the environment are called leaky or Black Queen Functions (BQF) [9]. As mentioned previously, a decrease in the selective pressure on genes encoding such functions will favour genome streamlining in some organisms which will begin to outgrow the microbes within the same population. In a homogeneous population, BQ states that loss-of-function (LOF) mutants will keep growing until an equilibrium between ancestral and mutant clones is reached, where they will compete for the same resources [9, 20]. Indeed, the mutant (beneficiary) will depend on the ancestral strain (helper) to complement the lost function, only if there are no other providers of the required good. In a real-life mixed microbial community, however, it is very likely that unrelated organisms can support the growth of the beneficiaries, without necessary competing for the same resources. In this case, the LOF mutant will take over the ancestral clone, engaging in a dependency relationship with unrelated helpers. It should be noted that, if requirements of helpers and beneficiaries are sufficiently disjointed, this relationship is rewarding for all the actors: beneficiaries can freely acquire the goods provided from other species, while the helpers can become necessary for the other species to thrive. The helper species is not affected by fluctuations of beneficiary species abundance, whereas a decrease of helpers would be detrimental for beneficiaries guarantee, on a community level, a shift from competition to coexistence [21, 22]: beneficiaries tend to be advantaged when they do not compete with their helpers, which means that nutritional specialization maximizes the resource allocation and the overall fitness of the community.

The importance of microbial communities for the environment, the geochemical cycles, and the health and development of coexisting eukaryotes is now acknowledged [23, 24]. More importantly, we know that microbial communities are “complex adaptive systems” [25], where individuals and populations interact, giving rise to the system's higher-order (emergent) properties; therefore, to understand the mechanisms underlying their composition is crucial. In this sense, BQ provides important evolutionary insights into the contribution of genome reduction to stratify dependency relationships within the community. For instance, the analysis of gut microbiota variability highlighted the presence of dominant alternative community compositions (enterotypes) [26], whose origin and nature are still debated [27–29]. A recent study [30] linked the emergence of different enterotypes to a group of strong interacting species, which are species groups characterized by a strong association. Stated differently, according to this model, patterns of association, or codependence, drive the community to different compositions with similar stability. As pointed out by the authors, this knowledge paves the way for translational applications into human health, in that the manipulation (i.e., addition or removal) of these strong interacting species can be used to alter the microbiome composition from unhealthy to healthy enterotypes.

In perspective, the technological advances of metagenomics, taking us closer to a “strain-level” resolution [31], will allow the integration of microbial ecology with evolutionary genomics. Thanks to advancements in dynamical modeling, it is possible to infer ecological interactions between species by measuring variations in abundances from metagenomics longitudinal data. For instance, Steinway et al. constructed a Boolean dynamic model from time series metagenomics data and used it to identify competitors of *Clostridium difficile,* using metabolic network reconstruction to break down the metabolic interactions occurring between microbial species [32]. Having the genome sequences, it will be possible to understand the evolutionary trajectories and the ecological interactions of the microbial communities. System biology approaches like constraint-based metabolic modeling, applied at a community level, will facilitate the knowledge-driven engineering of consortia, paving the way for a synthetic ecology [33].

## 3. Rising Complexity: Coevolving with Eukaryotic Hosts

An important factor related to reductive evolution of symbiotic microbes is the intimacy of the symbiotic relationship (obligate vs. facultative) with their eukaryotic hosts [34, 35]. For instance, obligate intracellular symbionts, such as *Buchnera aphidicola*, live in a nutritionally rich environment, with relatively low population size and little (if any) access to foreign DNA to acquire via Horizontal Gene Transfer (HGT). Thus, not surprisingly, this bacterium gradually accumulates inactivating mutation on “dispensable” genes which are successively lost [36, 37]. Again, as adaptive genome streamlining is shaped on the nutritional requirements of the symbiont, it is possible to predict the degree of the reduction, as well as, to some extent, the order of gene deletion [38]. On the other hand, bacteria engaging a less “radical” lifestyle, i.e., extracellular symbionts, are subject to a number of constraints including ecological interactions, fluctuation of nutrients, and dynamic changes of the community composition. Therefore, the evolution of these bacteria is less constrained, and their genome size can either increase or decrease, also depending on their lifestyle [34]. Finally, free-living bacteria able to colonize different niches, such as representatives of the genera *Burkholderia* and *Sinorhizobium*, are characterized by large genomes made up by multiple chromosomes and a notable phenotypic versatility which allows them to survive in different environments. It should be noted that, although being less common, reductive evolution also occurs among free-living bacteria [39].

The eukaryotic hosts are not only the environment in which the microbiota resides; they also coevolved with their symbionts, to the point that the microbiota exerts a huge influence over their health and development. Hosts have specific traits which favour microbes with beneficial effects for their health: for instance, epithelial cells in the human intestine modify their glycans to expose fucose [40], a sugar used by commensal bacteria which protect their host from pathogens and decrease inflammation. Similarly, plant roots produce exudates [41] which have a role in establishing the symbiosis with soil bacteria. By modulating the mechanisms promoting synthropic interactions in different districts within the host, different groups of microbes sharing metabolic connections (i.e., microbial guilds [42]) are established. Interestingly, in humans, LOF variants of genes responsible for the interaction with the microbiome are associated with pathogenic phenotypes. For instance, such variants in the gene FUT2, involved in the fucosilation of glycans, are associated with alterations in the gut microbiome, Crohn's disease, and diabetes [43–45].

Therefore, the genetic landscape of the host (along with other “environmental” factors such as lifestyle, diet, and infections) plays an important role in the selection and maintenance of the microbiome, which then influences the health and development of its host. Notwithstanding in the last decades, for some of these altered microbiota, a treatment has been possible by inoculating microbial mixtures obtained from the stool of healthy donors, a practice known as faecal microbial transplant (FMT) [46]. Although conceptually FMT is not different from classical probiotics (such as sour milk [47]), it poses the basis for a more focused approach, called bacteriotherapy, in which precise combinations of commensal microbes are provided to restore the microbiota to a balanced state [48]. A rational design of bacterial mixtures to be used as treatment requires not only the knowledge of the patient microbiome composition but also predictive models to infer the combination of strains able to restore the native microbiome functionalities.

## 4. Conclusions

In the last year, a rising number of evidences supported the evolutionary importance of reduction, rather than amplification, of genome size [13]. For prokaryotes, genome reduction is also coupled with an actual simplification, in terms of organism complexity. Morris proposed, with the BQ, that genome reduction comes not from neutral selection but is adaptive and strictly related to the leakiness properties in some biological functions. Currently, the analysis of the “social” interactions between microbes is shifting from monospecies populations of model organisms to complex entities such as microbial communities, modeled as economical systems to predict their time-resolved evolution [49, 50].

Here, we reviewed the ecological implications of genome streamlining in complex microbial systems. Although this mode of evolution has probably played a key role in shaping eukaryotic genomes [51], its impact on prokaryotes is perhaps even greater, with genome reduction directly influencing the emergence within bacterial communities of cooperation and cross-feeding patterns, which in turn affect the genome streamlining dynamics. Such ecological interactions are a primary force driving the composition of these systems: it is crucial to understand the behaviour and the composition dynamics of microbial communities to take into account the emergent constraints of cooccurrence between different species. Although the concept of “leaky function” is rather vague, the establishment of microbial guilds/consortia is primarily driven by nutritional and metabolic interactions.

As a concluding remark, we anticipate that it will be possible to achieve a “quantitative” understanding of the microbial ecology, thanks to the theoretical (e.g., reconstruction algorithms) and technical advancements of comparative genomics and metagenomics: indeed, the identification of metabolic pathways under purifying selection will allow to identify the nutritional constraints under which the organisms within the community are subject. This knowledge will be crucial to efficiently program alterations of the microbial ecology to drive the properties of microbial communities towards desired outcomes.
